# Assessment of inequalities in women’s and children’s health: Why, how, and for whom? Analyses of the Brazilian Live Birth Information System.

**DOI:** 10.1590/1980-5497202500XX

**Published:** 2025-05-30

**Authors:** Fernando César Wehrmeister, Janaína Calu Costa, Luiza Eunice Sá da Silva, Natalia Peixoto Lima, Francine dos Santos Costa, Aluísio Jardim Dornellas de Barros

**Affiliations:** I Universidade Federal de Pelotas, International Center for Equity in Health, Graduate Program in Epidemiology – Pelotas (RS), Brazil.; II Universidade de São Paulo, School of Public Health, Department of Epidemiology – São Paulo (SP), Brazil.

**Keywords:** Health inequality monitoring, Epidemiologic measurements, Live birth, Prenatal care

## Abstract

In this manuscript we discuss the importance of monitoring the analyses on health inequalities in women’s and children’s health. Data from the Brazilian Live Birth Information System were used to introduce the main inequality measures. The years from 2020 to 2022 were combined and at least one and eight or more antenatal care visits were considered as outcomes. As inequality measures, simple (ratios and differences) and complex measures for ordered (slope index of inequality and concentration index) and unordered (weighted and unweighted mean absolute difference from the mean) outcomes were presented. We discuss the strengths, limitations, and importance of inequality monitoring for researchers and policymakers.

## INTRODUCTION

Access to healthcare services is crucial for women’s and children’s health. The Constitution guarantees all Brazilians the right to health care through the Brazilian Unified Health System (SUS). However, systemic inequalities and inadequate resource allocation often hinder reaching all women and children, particularly in underserved areas and populations^1,2^. Leaving no women and children behind is key to a healthy society, and monitoring inequalities in women’s and children’s health-related outcomes is essential to achieve this.

In this study we aim to provide a simple guide to analyses of health inequalities, focusing on women’s and children’s health-related outcomes. Throughout the article, we present analyses as practical examples to demonstrate how to assess and interpret health disparities. All analyses relied on data from the Brazilian Live Birth Information System (*Sistema de Informações de Nascidos Vivos*–SINASC), which includes 99% of all live births in the country^3^. We combined individual data from 2020 to 2022 in a dataset comprising more than eight million live births.

## GETTING TO KNOW ITS OUTCOMES AND STRATIFIERS

The first step to conducting an inequality analysis is to recognize the nature of the outcomes and stratifiers.

In health-related outcomes or indicators, dichotomous variables represent the presence or absence of a health condition or behavior, the receipt of an intervention based on which the coverage or prevalence can be estimated. Alternatively, they can be continuous measures such as anthropometric indices, birth weight, etc.

Regarding stratifiers, the variables used to split our population into meaningful subgroups to assess the inequalities among them can differ. These variables may be dichotomous, with no specific order (such as sex and urban/rural area of residence), ordered (such as categories of wealth and educational attainment), or unordered variables (such as country’s regions and ethnic groups).

In this study we used two dichotomous indicators:

- coverage of at least one antenatal care visit (ANC);
- coverage of eight or more ANC visits.

As dimensions of inequalities, the stratifiers, we opted for women’s educational attainment in five groups (none, 1–3 years, 4–8 years, 8–11 years, and 12 or more years) and geographic region of residence (North, Northeast, Southeast, South, and Midwest).

## A GRAPHICAL APPROACH TO VISUALIZE AND IDENTIFY PATTERNS OF INEQUALITIES

The equiplot is an innovative way to visualize and identify patterns of inequalities (https://equidade.org/en/equiplot). In this graph, each dot represents the outcome of interest in a population subgroup, and the distance between them illustrates the magnitude of inequality.

**Figure 1:**
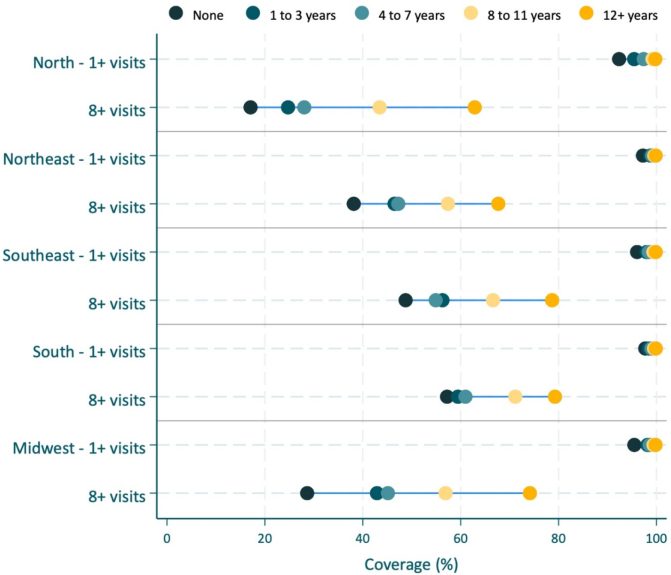
Coverage of at least one and eight or more antenatal care visits, according to women’s educational attainment by country regions. Brazil, Live Birth Information System, 2020–2022.

In Figure 1, we plotted our ANC indicators according to women’s educational attainment within each region. We can observe that at least one ANC visit coverage is 90% or higher for all subgroups in every region. Conversely, the coverage of eight or more ANC visits is much lower, and there is a huge gap between the uneducated and the more educated women, especially in the North and Midwest regions. Furthermore, there are inequalities between regions. The coverage of eight or more ANC visits for women with 8–11 years of formal education in the North region is lower than for women with none or 1–3 years of formal education in the South and Southeast regions.

In addition to visualizing the coverage by subgroup, monitoring patterns of inequalities can be helpful for policymakers and healthcare professionals. These patterns can be classified as linear (similar differences between groups), bottom (when the most vulnerable group is far behind the others), or top (when the less disadvantaged group is far ahead of the others) inequalities^4^. In Figure 1, we can identify the bottom inequality pattern in the North region for at least one ANC visit: while the uneducated women present coverage of around 90%, the other groups almost reached 100%. We also identified the top inequality pattern of eight or more ANC visits in the South and Southeast regions.

## ABSOLUTE OR RELATIVE MEASURES?

The next step is deciding whether you are interested in absolute or relative inequality measures. Due to mathematical artifacts, relative measures can be very high in rare events (i.e., Hepatitis C, a disease with an incidence of 1%). In contrast, absolute measures can be low when the event is frequent (i.e., institutional delivery coverage, above 95% in Brazil). It is recommended that both relative and absolute measures are presented, understanding the strengths and limitations of each on their calculations. However, depending on your audience, you may have to choose one to display. Usually, absolute measures are more straightforward to be understood by a broad audience.

## SIMPLE AND COMPLEX MEASURES OF INEQUALITIES

The third step is to calculate the inequality measure you are interested in. There are plenty of possible inequality measures^5^, and we will present those that we believe are easier to understand. To explain these calculations, in Table 1 we show the same data used to create Figure 1. Simple measures are the differences (absolute inequality) and ratios (relative inequality) calculated from the best to the worst performer regarding the outcome. We observed that the differences between the most educated and the uneducated women in eight or more ANC visits ranged from 22.1 percentage points (pp) in the South to 45.8 pp in the North, illustrating the magnitude of the inequality already observed in the figure. The ratios follow the same pattern, 1.39 and 3.68 times higher, for the most educated women compared to women with no formal education in the South and North regions, respectively (Table 1).

**Table 1:** Coverage of eight or more antenatal care visits, according to women’s educational attainment by country region. Brazil, Live Birth Information System, 2020-2022.

Region	Women’s educational attainment, complete years					Simple measures		Complex measures	
	None	1-3	4-7	8-11	12+	Difference (pp)	Ratio	SII (95%CI)	CIX (95%CI)
North	17.1	24.7	28.0	43.4	62.9	45.8	3.68	41.7 (41.3–42.1)	14.6 (14.5–14.7)
Northeast	38.2	46.5	47.2	57.4	67.7	29.5	1.77	24.5 (24.2–24.7)	6.6 (6.5–6.7)
Southeast	48.7	56.3	54.9	66.6	78.7	29.9	1.61	27.5 (27.3–27.7)	5.2 (5.2–5.2)
South	57.2	59.4	61.0	71.2	79.3	22.1	1.39	21.0 (20.7–21.3)	3.7 (3.6–3.8)
Midwest	28.6	42.9	45.1	56.9	74.1	45.5	2.59	36.4 (36.0–36.8)	8.3 (8.2–8.4)

pp: percentage points; 95%CI: 95% confidence interval; SII: slope index of inequality; CIX: concentration index.

Regarding interpretation, the higher the distance from values zero for differences and one for ratios, the higher the inequality. By convention, it is worth noting that positive values for differences and values above one for ratios indicate that the better-off group presents better performance. The opposite, for differences and ratios, means that the worst-performance group presents higher coverage or prevalence. This approach will also apply to complex measures for ordered groups.

The main advantage of complex measures of inequalities, compared to simple ones, is to account for the whole distribution of the outcome across the stratifier. The slope index of inequality (SII, absolute) and the concentration index (CIX, relative) are commonly used in inequality analyses for ordered stratifiers. The SII is based on regression (linear or logistic, depending on the outcome) and measures the distance between the predicted values at the extremes of the ordered stratifier^4-6^. The CIX uses a similar approach to the Gini index and measures how concentrated an outcome is according to an ordered stratifier. Both measures range from -1 to +1, with zero meaning the absence of inequality. As aforementioned, positive values mean the outcome is concentrated among the better-off group, while negative values mean the opposite. Multiplying the SII by one hundred for outcomes derived from a proportion is possible, expressing the difference as percentage points. CIX is a relative measure, and no unit can be attributed to it. If presenting SII and CIX together, multiplying both by one hundred could be easier for readers to understand. In Table 1, the SII ranges from 21.0 pp (95% confidence interval [CI] 20.7–21.3) in the South to 41.7 pp (95%CI 41.3–42.1) in the North, being the most unequal region in terms of pregnant women attending eight or more ANC visits. The CIX followed the same pattern as SII, which was higher in the North, accounting for 14.6 pp (95%CI 14.5–14.7) and lower in the South, with 3.7 pp (95%CI 3.6–3.8).

The mean absolute difference from the mean (MADM, weighted and unweighted, absolute measure) is the chosen complex measure for unordered groups. The idea behind this measure is to have an average difference from any reference value; this reference can be the national estimate or a target value for a specific intervention, such as BCG above 90%, which is the coverage target in Brazil. The unweighted MADM is the simple average of absolute difference values from the reference, assuming the same importance to the mean of all subgroups. The weighted MADM takes into account the subgroup share of the sample size to avoid higher values due to estimates from smaller groups, which could lead to an imprecise indicator estimate. The step-by-step calculations are shown in Table 2, which presents results on eight or more ANC visits by region and year. Using both weighted and unweighted MADM, we can observe that regional inequalities in Brazil reduced from 2020 to 2022, with the unweighted estimate being slightly higher than the weighted one.

**Table 2:** Coverage and inequality measures per country region by year. Brazil, Live Birth Information System, 2020-2022.

Year	Region	8+ antenatal care visits			Absolute difference between region and country estimates		MADM	
		%	Population	Population share (%)	Simple difference	Multiplied by the population share	Unweighted	Weighted
2020	North	37.9	288,648	0.108	22.1	2.4	9.9	9.0
	Northeast	52.7	738,362	0.277	7.3	2.0		
	Southeast	67.7	1,039,262	0.390	7.7	3.0		
	South	70.8	371,835	0.140	10.8	1.5		
	Midwest	58.6	226,850	0.085	1.4	0.1		
	*Brazil*	*60.0*	*2,664,957*					
2021	North	43.2	293,694	0.113	19.5	2.2	8.6	7.7
	Northeast	57.5	729,733	0.280	5.2	1.5		
	Southeast	69.1	998,642	0.383	6.4	2.4		
	South	72.7	358,591	0.138	10.0	1.4		
	Midwest	60.6	225,926	0.087	2.1	0.2		
	*Brazil*	*62.7*	*2,606,586*					
2022	North	47.1	279,295	0.111	17.4	1.9	7.3	6.2
	Northeast	61.0	686,709	0.273	3.5	1.0		
	Southeast	69.2	969,161	0.386	4.7	1.8		
	South	73.4	356,408	0.142	8.9	1.3		
	Midwest	62.5	222,245	0.088	2.0	0.2		
	*Brazil*	*64.5*	*2,513,818*					

MADM: Mean Absolute Difference from the Mean. The values for the entire country, which were used to calculate de MADM, are highlighted in italics.

## LIMITATIONS ON INEQUALITY MEASURES

The measures presented in this study, although commonly used, have some limitations. Simple measures are easier to understand, but do not account for the whole distribution in stratifiers with more than two categories. Moreover, estimating the 95%CI could not be straightforward in dichotomous outcomes, especially for differences, and bootstrapping strategies may be required to do it. Complex measures consider the entire distribution, but some issues should be accounted for. Based on generalized linear models, the SII should have a linear relationship between outcome and stratifier; otherwise, its use might be inappropriate. CIX can be applied only for positive values in the outcome — using indicators, such as anthropometric z-scores, is not possible. In addition, as a relative measure, CIX can be very high for rare outcomes and very low for common outcomes. The MADM is affected by the number of groups, and comparisons between different settings could not be straightforward. In this manuscript, we opted not to show a relative measure for unordered groups, as they are heavily affected by several factors, mainly the number of groups and the level of the outcome occurrence. Furthermore, any dispersion measure for MADM requires bootstrapping.

## CONCLUSIONS AND RECOMMENDATIONS

In this manuscript we presented some measures of inequality based on commonly-used stratifiers (level of education and country region), but several other dimensions can be explored. Additional analyses can explore intersectionality between groups and other essential domains such as women’s empowerment, gender, ethnicity, disability, etc. This choice will depend on how relevant a characteristic is in a given setting.

The choice of the appropriate inequality measure is crucial in communicating results to the target audience. The measure should allow distinguishing differences between subgroups and being understood by several actors, including academics, policymakers, healthcare professionals, and the general population.

The monitoring of health inequalities helps identifying differences among subgroups, providing feedback to healthcare systems on their organization and actions. Although the SUS is an example of a universal health system in the country, monitoring how health-related outcomes for women and children vary according to different subgroups is essential to leave no one behind, a key promise of the 2030 Sustainable Development Goals.
