# Supplementary material for: Assessment of inequalities in women’s and children’s health: Why, how, and for whom? Analyses of the Brazilian Live Birth Information System
Source: Rev Bras Epidemiol. 2025 May 30;28:e250029. doi: 10.1590/1980-5497202500XX (PMC12124669; doi:10.1590/1980-5497202500XX)
Supplement: Translation

# Translation: Avaliação das desigualdades na saúde de mulheres e crianças: por quê, como e para quem? Análises do Sistema de Informações sobre Nascidos Vivos do Brasil

**DOI:** 10.1590/1980-549720250029.2

**Authors:** Fernando César Wehrmeister, Janaína Calu Costa, Luiza Eunice Sá da Silva, Natalia Peixoto Lima, Francine dos Santos Costa, Aluísio Jardim Dornellas de Barros

**Affiliations:** I Universidade Federal de Pelotas, International Center for Equity in Health, Graduate Program in Epidemiology – Pelotas (RS), Brasil.; II Universidade de São Paulo, School of Public Health, Department of Epidemiology – São Paulo (SP), Brasil.

**Keywords:** Mensuração das desigualdades em saúde, Medidas em epidemiologia, Nascido vivo, Cuidado pré-natal

## Abstract

O presente manuscrito discute a importância de monitorar as análises das desigualdades em saúde de mulheres e crianças. Utilizamos dados do Sistema de Informações sobre Nascidos Vivos para apresentar as principais medidas de desigualdade. Combinamos os anos de 2020 a 2022 e utilizamos como desfecho a realização de pelo menos uma e oito ou mais consultas de prénatal. Como medidas de desigualdade, apresentamos medidas simples (razões e diferenças) e complexas para desfechos ordenados (índice absoluto de desigualdade e índice de concentração) e desfechos não ordenados (diferença absoluta média em relação à média, ponderada e não ponderada). Discutimos os pontos fortes, as limitações e a importância do monitoramento das desigualdades para pesquisadores e gestores de políticas públicas.

## INTRODUÇÃO

O acesso aos serviços de saúde é fundamental para a saúde de mulheres e crianças. A Constituição Federal assegura a todos os brasileiros o direito à assistência médica por meio do Sistema Único de Saúde (SUS). No entanto, desigualdades sistêmicas e a alocação inadequada de recursos frequentemente dificultam o acesso aos serviços para todas as mulheres e crianças, especialmente em áreas e populações vulneráveis^1,2^. Garantir que nenhuma mulher ou criança seja deixada para trás é essencial para a construção de uma sociedade saudável, sendo o monitoramento das desigualdades nos desfechos relacionados à saúde feminina e infantil crucial para alcançar esse objetivo.

Neste estudo, buscou-se oferecer um guia simples para a análise das desigualdades em saúde, com ênfase nos desfechos relacionados à saúde de mulheres e crianças. Ao longo do artigo, foram apresentados exemplos de análises que visam ilustrar como avaliar e interpretar as desigualdades em saúde. Todas as análises foram realizadas com base em dados do Sistema de Informações sobre Nascidos Vivos (SINASC), que abrange 99% dos nascidos vivos no país^3^. Foram combinados dados individuais de 2020 a 2022, resultando em um conjunto de dados que engloba mais de oito milhões de nascidos vivos.

## CONHECENDOS OS DESFECHOS E ESTRATIFICADORES

O primeiro passo na condução de uma análise de desigualdade é reconhecer a natureza dos desfechos e dos estratificadores.

Em desfechos ou indicadores relacionados à saúde, as variáveis dicotômicas representam a presença ou ausência de uma condição ou comportamento de saúde, ou o recebimento de uma intervenção, a partir da qual é possível estimar a cobertura ou prevalência. Alternativamente, essas variáveis podem ser contínuas, como índices antropométricos, peso ao nascer, entre outros.

Em relação aos estratificadores, as variáveis utilizadas para estratificar a população em subgrupos, a fim de avaliar as desigualdades entre eles, podem variar. Essas variáveis podem ser dicotômicas, sem ordem específica (como sexo e área de residência urbana/rural), ordenadas (como poder aquisitivo e nível educacional) ou não ordenadas (como regiões do país e grupos étnicos).

Neste estudo, foram utilizados dois indicadores dicotômicos:

- cobertura de pelo menos uma consulta de atenção pré-natal (APN);
- cobertura de oito ou mais consulta de APNs.

Como dimensões das desigualdades, os estratificadores adotados foram a escolaridade das mulheres, dividido em cinco grupos (nenhum, 1-3 anos, 4-8 anos, 8-11 anos e 12 ou mais anos), e a região geográfica de residência (Norte, Nordeste, Sudeste, Sul e Centro-Oeste).

## UMA ABORDAGEM GRÁFICA PARA VISUALIZAR E IDENTIFICAR PADRÕES DE DESIGUALDADES

O *equiplot*é uma abordagem inovadora para visualizar e identificar padrões de desigualdade (https://equidade.org/en/equiplot). Nesse gráfico, cada ponto representa o desfecho de interesse em um subgrupo populacional, e a distância entre eles indica a magnitude da desigualdade.

Na Figura 1, são apresentados os indicadores de APN estratificados por escolaridade das mulheres em cada região. Observa-se que a cobertura de pelo menos uma consulta de APN atinge 90% ou mais em todos os subgrupos e regiões. No entanto, a cobertura de oito ou mais consultas de APN é consideravelmente menor, com uma diferença expressiva entre mulheres com nenhum ano de estudo e aquelas com maior nível de escolaridade, especialmente nas regiões Norte e Centro-Oeste. Além disso, verificam-se desigualdades entre as regiões: na região Norte, a cobertura de oito ou mais consultas de APN para mulheres com 8 a 11 anos de escolaridade é inferior à observada para mulheres sem escolaridade ou com 1 a 3 anos de estudo nas regiões Sul e Sudeste.

**Figura 1:** Cobertura de pelo menos uma consulta e oito ou mais consultas de pré-natal, conforme o nível de escolaridade das mulheres por regiões do país. Brasil, Sistema de Informação sobre Nascidos Vivos, 2020–2022.

Além de visualizar a cobertura por subgrupo, o monitoramento dos padrões de desigualdade pode ser uma ferramenta valiosa para formuladores de políticas e profissionais de saúde. Esses padrões podem ser classificados como desigualdade linear (quando as diferenças entre os grupos são relativamente semelhantes), *bottom inequality* (quando o grupo mais vulnerável apresenta cobertura significativamente inferior aos demais) ou *top inequality* (quando o grupo menos desfavorecido apresenta cobertura substancialmente superior aos outros)^4^. Na Figura 1, observa-se um padrão *bottom inequality* na região Norte para pelo menos uma consulta de APN: enquanto as mulheres sem escolaridade apresentam uma cobertura em torno de 90%, os demais grupos alcançam quase 100%. Além disso, identifica-se um padrão *top inequality* para oito ou mais consultas de APN nas regiões Sul e Sudeste.

## MEDIDAS ABSOLUTAS OU RELATIVAS?

O próximo passo consiste em determinar se o interesse recai sobre medidas de desigualdade absoluta ou relativa. Devido a artefatos matemáticos, medidas relativas podem apresentar valores elevados em eventos raros (por exemplo, hepatite C, uma doença com incidência de 1%). Em contrapartida, medidas absolutas tendem a ser baixas quando o evento é frequente (por exemplo, a cobertura de parto institucional, que supera 95% no Brasil). Recomenda-se a apresentação simultânea de medidas relativas e absolutas, considerando os pontos fortes e as limitações inerentes a cada abordagem. No entanto, dependendo do público-alvo, pode ser necessário optar por apenas uma delas. Em geral, medidas absolutas são mais intuitivas e de fácil compreensão para um público mais amplo.

## MEDIDAS SIMPLES E COMPLEXAS DE DESIGUALDADES

O terceiro passo consiste no cálculo da medida de desigualdade de interesse. Existem diversas medidas possíveis^5^, e serão apresentadas aquelas que se mostram mais intuitivas. Para ilustrar esses cálculos, a Tabela 1 exibe os mesmos dados utilizados na construção da Figura 1. As medidas simples incluem as diferenças (desigualdade absoluta) e as razões (desigualdade relativa), calculadas a partir da comparação entre o melhor e o pior desempenho em relação ao desfecho analisado. Observa-se que as diferenças na cobertura de oito ou mais consultas de APN entre mulheres com maior nível de escolaridade e aquelas sem escolaridade variaram de 22,1 pontos percentuais (pp) na região Sul a 45,8 pp na região Norte, evidenciando a magnitude da desigualdade já identificada na figura. As razões seguem o mesmo padrão, sendo 1,39 e 3,68 vezes maiores para as mulheres com maior escolaridade em comparação às sem educação formal nas regiões Sul e Norte, respectivamente (Tabela 1).

**Tabela 1:** Cobertura de oito ou mais consultas de pré-natal, conforme o nível de escolaridade das mulheres por região do país. Brasil, Sistema de Informação sobre Nascidos Vivos, 2020-2022.

Região	Nível de escolaridade das mulheres, anos completos					Medidas simples		Medidas complexas	
	Nenhuma	1-3	4-7	8-11	12+	Diferença (pp)	Razão	SII (IC95%)	CIX (IC95%)
Norte	17,1	24,7	28,0	43,4	62,9	45,8	3,68	41,7 (41,3–42,1)	14,6 (14,5–14,7)
Nordeste	38,2	46,5	47,2	57,4	67,7	29,5	1,77	24,5 (24,2–24,7)	6,6 (6,5–6,7)
Sudeste	48,7	56,3	54,9	66,6	78,7	29,9	1,61	27,5 (27,3–27,7)	5,2 (5,2–5,2)
Sul	57,2	59,4	61,0	71,2	79,3	22,1	1,39	21,0 (20,7–21,3)	3,7 (3,6–3,8)
Centro-Oeste	28,6	42,9	45,1	56,9	74,1	45,5	2,59	36,4 (36,0–36,8)	8,3 (8,2–8,4)

pp: pontos percentuais; IC95%: intervalo de confiança de 95%; SII: índice absoluto de desigualdade (*slope index of inequality*); CIX: índice de concentração (*concentration index*).

Quanto à interpretação, quanto maior a distância em relação ao valor zero para as diferenças e ao valor um para as razões, maior é a desigualdade observada. Por convenção, valores positivos para as diferenças e valores superiores a um para as razões indicam que o grupo com melhor desempenho apresenta maior cobertura ou prevalência. O inverso também é válido: valores negativos para as diferenças e inferiores a um para as razões sinalizam que o grupo com pior desempenho apresenta maior cobertura ou prevalência. Essa abordagem também se aplica a medidas complexas utilizadas para grupos ordenados.

A principal vantagem das medidas complexas de desigualdade, em comparação com as medidas simples, é a consideração de toda a distribuição do desfecho dentro do estratificador. O índice absoluto de desigualdade (*slope index of inequality*– SII, absoluto) e o índice de concentração (*concentration index*– CIX, relativo) são amplamente utilizados em análises de desigualdade para estratificadores ordenados. O SII baseia-se em regressão (linear ou logística, dependendo do desfecho) e mede a distância entre os valores previstos nos extremos do estratificador ordenado^4–6^. O CIX, por sua vez, adota uma abordagem semelhante ao índice de Gini e avalia o grau de concentração de um desfecho em relação a um estratificador ordenado. Ambas as medidas variam de -1 a +1, sendo que zero indica ausência de desigualdade. Conforme mencionado anteriormente, valores positivos indicam maior concentração do desfecho no grupo mais favorecido, enquanto valores negativos indicam o oposto. Para desfechos expressos em proporções, o SII pode ser multiplicado por cem, permitindo sua interpretação em pontos percentuais. O CIX, sendo uma medida relativa, não possui unidade atribuída. Quando SII e CIX são apresentados conjuntamente, multiplicá-los por cem pode facilitar a compreensão dos leitores. Na Tabela 1, o SII variou de 21,0 pp (intervalo de confiança [IC] de 95% 20,7–21,3) na região Sul a 41,7 pp (IC95% 41,3–42,1) na região Norte, indicando que essa última apresenta a maior desigualdade em relação à cobertura de oito ou mais consultas de APN. O CIX seguiu o mesmo padrão do SII, sendo maior no Norte, com 14,6 pp (IC95% 14,5–14,7), e menor no Sul, com 3,7 pp (IC95% 3,6–3,8).

A diferença absoluta média em relação à média (*mean absolute difference from the mean*– MADM, ponderada e não ponderada, medida absoluta) foi a medida complexa escolhida para grupos não ordenados. O conceito por trás dessa medida é calcular uma diferença média em relação a qualquer valor de referência; essa referência pode ser a estimativa nacional ou um valor alvo para uma intervenção específica, como a cobertura da vacina BCG, cuja meta no Brasil é superior a 90%. O MADM não ponderado é a média simples das diferenças absolutas em relação à referência, assumindo a mesma importância para todos os subgrupos ao calcular a média. Por outro lado, o MADM ponderado leva em consideração a participação de cada subgrupo no tamanho da amostra, evitando que estimativas de grupos menores influenciem excessivamente o valor final, o que poderia resultar em uma estimativa imprecisa do indicador. Os cálculos detalhados passo a passo estão apresentados na Tabela 2, que mostra os resultados para oito ou mais consultas de atenção pré-natal por região e ano. Ao utilizar tanto o MADM ponderado quanto o não ponderado, observa-se que as desigualdades regionais no Brasil diminuíram entre 2020 e 2022, com a estimativa não ponderada sendo ligeiramente superior à ponderada.

**Tabela 2:** Cobertura e medidas de desigualdade por região do país e por ano. Brasil, Sistema de Informação sobre Nascidos Vivos, 2020-2022.

Ano	Região	8+ consultas de pré-natal			Diferença absoluta entre a estimativa da região e do país		MADM	
		%	População	Participação da população (%)	Diferença simples	Multiplicada pela participação da população	Não ponderada	Ponderada
2020	Norte	37,9	288.648	0,108	22,1	2,4	9,9	9,0
	Nordeste	52,7	738.362	0,277	7,3	2,0		
	Sudeste	67,7	1.039.262	0,390	7,7	3,0		
	Sul	70,8	371.835	0,140	10,8	1,5		
	Centro-Oeste	58,6	226.850	0,085	1,4	0,1		
	*Brasil*	*60,0*	*2.664.957*					
2021	Norte	43,2	293.694	0,113	19,5	2,2	8,6	7,7
	Nordeste	57,5	729.733	0,280	5,2	1,5		
	Sudeste	69,1	998.642	0,383	6,4	2,4		
	Sul	72,7	358.591	0,138	10,0	1,4		
	Centro-Oeste	60,6	225.926	0,087	2,1	0,2		
	*Brasil*	*62,7*	*2.606.586*					
2022	Norte	47,1	279.295	0,111	17,4	1,9	7,3	6,2
	Nordeste	61,0	686.709	0,273	3,5	1,0		
	Sudeste	69,2	969.161	0,386	4,7	1,8		
	Sul	73,4	356.408	0,142	8,9	1,3		
	Centro-Oeste	62,5	222.245	0,088	2,0	0,2		
	*Brasil*	*64,5*	*2.513.818*					

MADM: diferença absoluta média em relação à média (*Mean Absolute Difference from the Mean*). Os valores para o país como um todo, usados para calcular o MADM, estão destacados em itálico.

## LIMITAÇÕES NAS MEDIDAS DE DESIGUALDADE

As medidas apresentadas neste estudo, embora amplamente utilizadas, apresentam algumas limitações. As medidas simples são mais fáceis de entender, mas não consideram toda a distribuição quando o estratificador possui mais de duas categorias. Além disso, a estimativa do IC de 95% pode não ser simples em desfechos dicotômicos, especialmente no caso das diferenças, sendo necessárias estratégias de *bootstrapping* para realizá-la adequadamente. As medidas complexas, por sua vez, levam em conta toda a distribuição, mas também apresentam desafios. No caso do SII, que é baseado em modelos lineares generalizados, deve existir uma relação linear entre o desfecho e o estratificador; caso contrário, seu uso pode não ser adequado. O CIX pode ser aplicado apenas para desfechos positivos, não sendo possível utilizá-lo para indicadores como escores z antropométricos. Além disso, por ser uma medida relativa, o CIX pode apresentar valores elevados para desfechos raros e muito baixos para desfechos comuns. O MADM é influenciado pelo número de grupos, o que torna as comparações entre diferentes configurações complexas. Neste manuscrito, optou-se por não apresentar uma medida relativa para grupos não ordenados, pois elas são fortemente impactadas por vários fatores, como o número de grupos e o nível de ocorrência do desfecho. Além disso, qualquer medida de dispersão para o MADM exige o uso de *bootstrapping*.

## CONCLUSÕES E RECOMENDAÇÕES

Neste manuscrito, foram apresentadas algumas medidas de desigualdade com base em estratificadores comumente utilizados (escolaridade e região do país), mas diversas outras dimensões podem ser exploradas. Análises adicionais podem investigar a interseccionalidade entre grupos e outros domínios essenciais, como empoderamento feminino, gênero, etnia, deficiência, entre outros. A escolha dessas dimensões dependerá da relevância de cada característica em um contexto específico.

A escolha da medida de desigualdade adequada é fundamental para a comunicação eficaz dos resultados ao público-alvo. A medida selecionada deve possibilitar a distinção entre as diferenças entre subgrupos e ser de fácil compreensão para diversos atores, incluindo acadêmicos, formuladores de políticas, profissionais de saúde e a população em geral.

O monitoramento das desigualdades em saúde é fundamental para identificar diferenças entre subgrupos, oferecendo *feedback* aos sistemas de saúde sobre sua organização e ações. Embora o SUS seja um exemplo de sistema de saúde universal no Brasil, é essencial monitorar como os desfechos relacionados à saúde de mulheres e crianças variam entre diferentes subgrupos para garantir que ninguém seja deixado para trás, conforme prometido nos Objetivos de Desenvolvimento Sustentável de 2030.
